# Surgical Management of Metastatic Extraskeletal Extradural Ewing Sarcoma of the Thoracic Spine in a Patient with Wiskott–Aldrich Syndrome and Hypertrophic Cardiomyopathy: A Case Report

**DOI:** 10.1055/a-2914-8018

**Published:** 2026-07-27

**Authors:** Kent Sabatose, Daniel Wiggan, Suzan De Feo, Erinc Akture, Wissam Elfallal

**Affiliations:** 1 Department of Neurosurgery 70047 College of Medicine, Lake Erie College of Osteopathic MedicineBradentonFloridaUnited States; 2 Department of NeurosurgeryNew York University Grossman School of MedicineNew York CityNew YorkUnited States; 3 Department of NeurosurgeryAdventHealth Neuroscience InstituteDaytona BeachFloridaUnited States; 4 Department of NeurosurgeryIstanbul University-CerrahpaşaIstanbulTürkiye; 5 Department of NeurosurgeryBaptist Neurological InstituteJacksonvilleFloridaUnited States; 6 Department of Neurosurgery 6918 Oakland University William Beaumont School of MedicineRoyal OakMichiganUnited States

**Keywords:** Ewing sarcoma, extraskeletal Ewing sarcoma, Wiskott–Aldrich syndrome, oncology

## Abstract

**Background** Wiskott–Aldrich syndrome (WAS) is an X-linked recessive disorder increasing the risk of malignancy, commonly lymphoma. Extraskeletal Ewing sarcoma (EES) is rare, typically occurring in the trunk, extremities, and retroperitoneum. Features of EES include rapid growth, quick metastasis, and localized pain.

**Observations** A 31-year-old male with a history of WAS and hypertrophic cardiomyopathy (HCM) reported to the emergency department for a 1-day onset of difficulty ambulating, bilateral lower extremity weakness, numbness ascending to the thorax, and mid-back pain. A T5 sensory level was established with genitalia sensation and bulbocavernosus reflexes absent. Magnetic resonance imaging (MRI) with and without contrast of the cervical, thoracic, and lumbar spine revealed a contrast-enhancing mass with extraosseous extension producing severe spinal stenosis and cord compression.

Acute neurological deterioration necessitated emergency decompression. Intraoperative pathology showed a small blue-cell tumor concerning for neuroectodermal tumor. Total resection was attempted with T1–T3 fusion and right T2 transpedicular costotransversectomy. Posttumor debulking, ambulation and sensation were regained. Pathological specimens were consistent with EES.

**Lessons** WAS increases the risk for lymphoma, though broad differentials should be considered, as neurosurgical debulking may be required for neurological recovery in non-radiosensitive tumors. HCM and WAS increase intraoperative complexity and the need for interdisciplinary collaboration in oncological therapy.

## Background

Wiskott–Aldrich syndrome (WAS) is an X-linked syndrome characterized by thrombocytopenia, eczema, and recurrent infections. 1 The mainstay of treatment for WAS is hematopoietic stem cell transplantation (HSCT), which the patient in our case received as a child. Other treatments, including symptomatic medical management of eczema, thrombocytopenia, and infections, are often required. 1 2 3 4 Lymphoma, specifically Epstein–Barr virus-positive B-cell lymphoma, has a strong association with WAS, occurring in approximately 13% of patients with the condition. 3 Patients with WAS who have undergone HSCT may be at greater risk for developing malignancy later in life, which may have contributed to our patient’s disease process. 5

Ewing sarcoma (ES) tumors are a group of small round cell tumors, including ES of the bone (ESB), peripheral primitive neuroectodermal tumor, a tumor of the chest wall, and extraskeletal Ewing sarcoma (EES). 6 7 8 ESB, the second most common pediatric primary tumor of bone, has an incidence approximately 10 times that of EES. 8 Approximately 40 cases of extradural EES have been reported in the literature, with most occurring in male adolescents and young adults and treated with surgical excision, radiation, and chemotherapy. 9 To our knowledge, there has been no report of EES occurring in patients with WAS or in patients with comorbid hypertrophic cardiomyopathy (HCM), complicating surgical and medical management.

We present a case of extradural EES in a patient with WAS presenting with rapid-onset ascending paralysis and anesthesia treated with emergent neurosurgical decompression. Surgical planning complexity was observed due to the presence of HCM.

## Observations

A 31-year-old male with a past medical history of WAS, central nervous system (CNS) lymphoma, HCM, and severe WAS-associated eczema presented to the emergency department with a chief complaint of progressing bilateral lower extremity weakness, back pain, and numbness ascending from his feet to his chest approximately 2 hours prior to seeking care.

Neurological exam showed cranial nerves (CNs) II–XII intact except left CN II; no pupillary light response due to history of CNS lymphoma. Full strength (5/5) and 2+ deep tendon reflexes (DTRs) noted in bilateral upper extremities. Light touch, proprioception, and nociception sensations are intact in the bilateral upper extremities. Bilateral absent sensation to light touch and pinprick below the nipple line (T5) is more pronounced on the left. The umbilical reflex was absent from all quadrants. Absent muscle strength (0/5) in the left psoas, quadriceps femoris, extensor hallicus longus (EHL), and dorsiflexion, and 1/5 strength with plantar flexion. Right-sided quadriceps femoris strength 1/5, dorsiflexion 2/5, EHL 3/5, and plantar flexion 4-/5. Absent genitalia sensation, bulbocavernosus reflexes, and rectal tone. Babinski and Hoffman reflexes are absent bilaterally. DTRs +1 in bilateral lower extremities throughout.

Vitals showed hypertension (168/91 mm Hg), tachycardia (124 beats/min), respirations (11 breaths/minute), afebrile, and 100% peripheral oxygen saturation. Laboratory analysis, including complete blood count, basic metabolic panel (BMP), coagulation studies, C-reactive protein, sedimentation rate, and lactic acid, was unremarkable except for leukocytosis 12.27 × 10 ^3^ /μL (77.3% segmented neutrophils).

Magnetic resonance imaging (MRI) of the cervical, thoracic, and lumbar spine with and without contrast was performed. MRI of the cervical spine showed (1) no evidence of acute osseous abnormality; (2) degenerative disk changes with disk protrusions at C5–6 and C6–7, producing flattening of the ventral cord. There was no significant spinal stenosis or neural foraminal narrowing. (3) There was a presence of a heterogeneous nodular thyroid gland. The MRI of the thoracic spine ( Fig. 1 ) showed (1) a mass centered along the posterior arch of T2 with extraosseous extension, effacing the thecal sac and producing severe spinal stenosis with cord compression. There was no significant edema within the adjacent cord. As there was an additional lesion within the sacrum, metastatic disease was considered. (2) There was an extension through the right neural foramen of T2–3 with contrast enhancement of the right costovertebral junction. (3) There was mild T2 hyperintensity with contrast enhancement of the T2 vertebral body and spinous process representing reactive changes, but infiltration of the osseous structures could not be excluded. (4) No evidence of acute fracture or subluxation. (5) Mild degenerative changes. MRI of the lumbar spine showed (1) no evidence of acute osseous abnormality. (2) Milder degenerative changes without significant spinal stenosis. Neural foraminal narrowing was as indicated above. (3) No evidence of abnormal contrast enhancement within the conus or cauda equina. (4) There were lesions within the L5 vertebral body, the S2 and S3 vertebral bodies, along the left sacral wing concerning for metastatic disease.

**Fig. 1:**
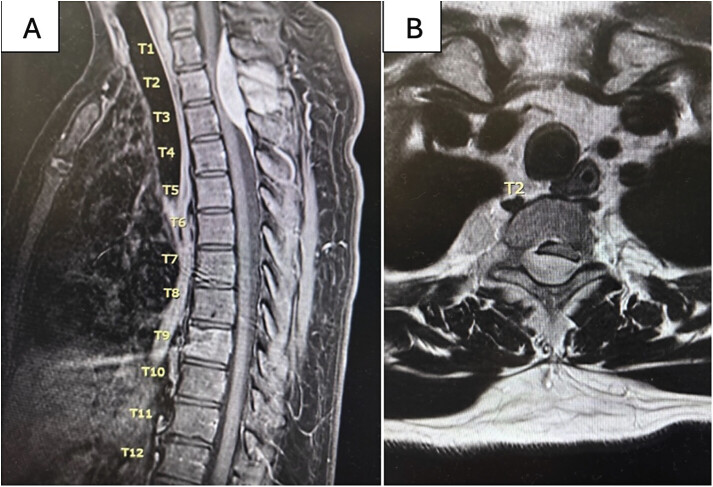
T1-weighted MRI with contrast showing sagittal ( **A** ) and axial ( **B** ) views of a contrast-enhancing extradural mass extending from T1 to T4 with extraosseous extension. Note the spinal canal stenosis with cord compression in ( **B** ) at the T2 level. MRI, magnetic resonance imaging.

Dexamethasone 10 mg injection was administered prior to transfer to our institution for emergency decompression with thoracic T1–T3 fusion with right T2 transpedicular costotransversectomy with resection of epidural mass ( Fig. 2 ). The patient was classified as an American Society of Anesthesiologists (ASA) 3, with total intravenous general anesthesia planned following cardiac clearance. Cardiac workup discovered a new diagnosis of HCM. Presurgical planning between neuroanesthesiology and neurosurgery was performed to consider the risk of general anesthesia with cardiac risk, and the need for thoracic spinal cord decompression. Surgery was scheduled with intraoperative neuromonitoring of bilateral upper and lower extremities, intraoperative ultrasound, and intraoperative fluoroscopy with C-arm. Intraoperative HCM precautions included patient positioning in the Trendelenburg position, arterial line placement for continuous blood pressure monitoring, and central line placement for large-volume fluid administration to maintain elevated heart preload throughout the procedure to ensure adequate spinal cord perfusion intraoperatively.

**Fig. 2:**
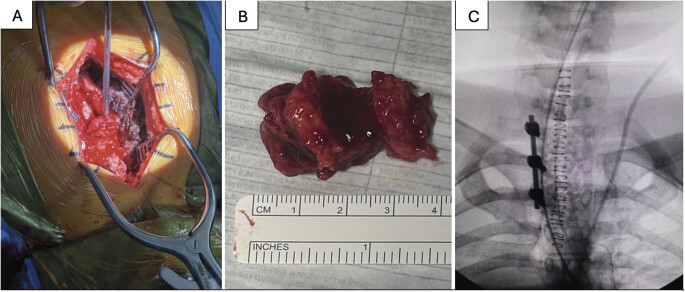
Intraoperative view showing extradural mass lifting from dura mater ( **A** ), with mass resected shown in ( **B** ). Postoperative plain radiograph of costotransversectomy and mass resection showing hardware from T1 to T3 unilateral fusion ( **C** ).

During the procedure, a large mass was found to be emanating from the bottom of the T1–T3 in the interspinous space as well as in the paraspinal musculature extending to the right facet complex. A biopsy of the mass was sent for frozen section, resulting in a diagnosis of possible small-cell or metastatic carcinoma. It was noted to lack characteristics of lymphoma, so complete resection was attempted. Laminectomies of T1, T2, and T3 were then performed. The tumor was then dissected, and any residual components were removed, requiring a transpedicular approach to T2, along with transection of T1 and T2 nerve roots. Unilateral fusion was performed from T1 to T3. Final surgical diagnosis was metastatic carcinoma epidural mass with extraosseous extension and posterior element involvement.

Metastatic workup was completed, including chest, renal ultrasound, and computed tomography (CT) of the chest, abdomen, pelvis, and brain. The chest X-ray showed mild bilateral central pulmonary vascular congestion. The renal ultrasound showed (1) a 6.8 × 6.3 × 6.3 cm exophytic solid and cystic mass in the lower pole of the right kidney, concerning primary renal malignancy; (2) mild right hydronephrosis; (3) increased bilateral renal cortical echogenicity; (4) an indeterminate 4.7 × 4.4 × 4 cm hypoechoic lesion in the spleen. Cardiology evaluated the patient, and an echo showed severely increased left ventricular wall thickness consistent with concentric hypertrophy. Ejection fraction estimated at 60 to 65%. Severe left ventricular outflow tract (LVOT) obstruction at rest. Normal right ventricular systolic function.

On postoperative day (POD) 1, neurologic exam demonstrated an increase in left lower extremity motor strength rated 1/5 throughout all muscle groups. Right lower extremity hip flexor strength increased to 2/5, and knee flexor and extensor strength increased to 3/5. Dorsiflexion and plantarflexion remained at 4/5 strength. Pressure and proprioception sensation improved from the T5 level; however, proprioception remained absent from the bilateral feet. Pinprick sensation remained absent up to the T5 sensory level. POD 2–4, the patient continued to show improved sensations and strength in his lower extremities. Left knee flexors and extensors rated 3/5, and dorsiflexion, plantar flexion, inversion, and eversion rated 4/5. No change in the right lower extremity strength. The pathology report of the soft tissue thoracic spine epidural mass was a poorly differentiated malignancy, consistent with ES. The patient was discharged on POD 4 with a referral to a sarcoma specialty center.

At an outside institution, the patient underwent radiation therapy with neoadjuvant chemotherapy with vincristine, doxorubicin, and cyclophosphamide/ifosfamide and etoposide (VDC/IE) protocol. He also underwent an en bloc resection of the right retroperitoneal mass consistent with EES, along with right nephrectomy. Despite these measures, metastatic disease progressed, resulting in the patient presenting approximately 1 year later with chief complaints of urinary incontinence and lumbar pain. He also presented with normocytic normochromic anemia with a hemoglobin of 5.5 and platelets at 49 × 10 ^9^ /L.

MRI of the lumbar and thoracic spine with and without contrast was notable for diffuse osseous and extraosseous metastatic disease, most pronounced at T5, associated with a pathological compression fracture and extraosseous extension with spinal cord compression ( Fig. 3 ). The patient was deemed unfit for surgical intervention due to the presence of persisting anemia and thrombocytopenia despite 2 units of packed red blood cells and 1 unit of platelets. The patient was recommended for consultation for proton beam therapy and to explore clinical trials available for his condition through his oncology team. He was discharged on a steroid taper.

**Fig. 3:**
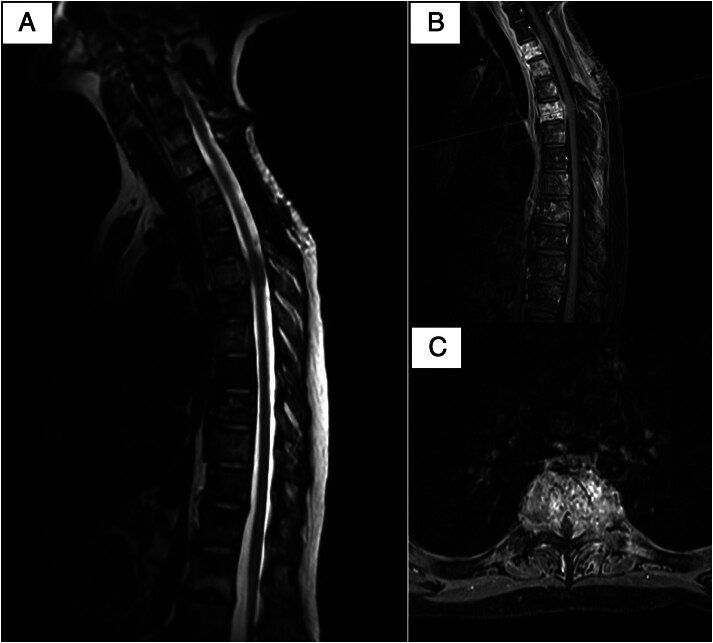
Approximately 1 year postoperatively, ( **A** ) T2-weighted MRI showing metastatic disease involving the cervical, thoracic, and lumbar spine. ( **B** ) Sagittal T1-weighted MRI with contrast of the thoracic spine showing contrast enhancement most significant at T5 (blue line) associated with a pathological compression fracture, extraosseous extension, and spinal cord compression. ( **C** ) Axial T1-weighted MRI with contrast at the T5 level showing a contrast-enhancing lesion causing spinal cord compression. MRI, magnetic resonance imaging.

## Discussion

ES is a highly aggressive small round cell tumor that predominantly affects younger individuals, with most cases diagnosed between 10 and 20 years of age. It arises primarily in bone but can also occur in soft tissues, as in this case. It is characterized by the translocation t(11;22)(q24;q12), resulting in fusion of the *EWSR1* gene on chromosome 22 with the *FLI1* gene on chromosome 11, a defining molecular event that underpins tumorigenesis. 10 11 In addition to molecular confirmation, immunohistochemical staining of CD99 remains a critical adjunct in establishing the diagnosis of ES in challenging or atypical presentations. 12 13 The growing role of genetic testing further supports accurate diagnosis and may help refine risk stratification and therapeutic decision-making in ES. 12 In our case, urgent surgical debulking was utilized due to radiographically and clinically confirmed neurological compromise with progressing symptoms, which ultimately led to the diagnosis of EES with a pathological specimen.

A multidisciplinary approach, as seen in our case, is commonly used in the treatment of EES due to the involvement of multiple body systems and comorbidities that complicate management. Surgical resection, chemotherapy, and sometimes radiation therapy are the hallmarks of ES treatment. 10 11 For localized disease, chemotherapy with vincristine, doxorubicin, cyclophosphamide/ifosfamide, etoposide (VDC/IE) has significantly improved prognosis, allowing for a 5-year survival rate of approximately 70 to 80%. 14 Surgical decision-making in our case was multifactorial, considering the patient’s medical history of HCM, the presence of undiagnosed soft tissue and bony lesions throughout the spine, pelvis, and skull, and the severe compression of the thoracic spinal cord. Surgical debulking was planned for the thoracic lesion only, as the other lesions presented no radiographic evidence of neurological compromise, and the patient’s exam was consistent with a sensory level corresponding to the thoracic lesion location. Once the intraoperative frozen specimen ruled out lymphoma, which was initially suspected due to the history of WAS, gross total resection with thoracic spinal cord decompression became the primary focus for proceeding. Vertebrectomy was a consideration to achieve gross total en bloc resection, though the risk of blood loss reducing preload in the setting of HCM outweighed the benefit, as it was not a solitary lesion. 15 Complete debulking was attempted utilizing a transpedicular approach to gain appropriate access to the rightward ventrolateral position of the tumor on the thoracic spinal cord. Due to the tight space associated with the thoracic spinal canal, a transpedicular approach along with transection of T1–T2 nerve roots allowed for mobilization of the thoracic cord to achieve maximal tumor resection. 16

Due to the intraoperative evidence of structural compromise of the posterior column secondary to tumor dissection, the need for fusion was assessed through the Spinal Instability Neoplastic Score (SINS) and the Neurologic, Oncologic, Mechanical, and Systemic (NOMS) framework. 17 18 19 20 21 Adjuvant radiation therapy associated with potential further structural destabilization also increased the need for unilateral fusion in our patient. 22

Postoperatively, the patient experienced significant neurological recovery to the point of independent ambulation. Despite aggressive multidisciplinary therapy, including surgical resection, VDC/IE therapy, and radiation, our patient with EES continues to demonstrate progression in tumor burden. This is consistent with poorer prognosis associated with higher stage at diagnosis and may also suggest the presence of additional oncogenic factors in EES, particularly in the setting of underlying genetic immunodeficiency. 23 24

## Conclusion

ES can arise extraskeletally and lead to multisystem metastatic disease. EES is aggressive and must be treated with targeted immunotherapies, radiation, and surgical resection when leading to mass effect. WAS is associated with an increased risk of malignancies, including EES. Symptoms of EES may arise suddenly if the affected area is sensitive to volume changes, such as the thoracic spinal canal. Emergency surgical decompression of the thoracic spine can be performed safely on a patient with known HCM when appropriate planning between neuroanesthesiology and the neurosurgeon is conducted.

Case reports are inherently limited by a single subject and a retrospective nature. Our patient continues to receive treatment for EES at an outside institution without plans for ongoing follow-up. The tumorigenesis among patients with WAS and those who have undergone HSCT is limited largely to lymphoma in the literature. It should be expanded to include other metastatic diseases, such as EES, due to the aggressive nature seen in our case, as well as the poor response to available targeted therapies.
